# Isolation and Characterization of Bacterial Endophytes from Small Nodules of Field-Grown Peanut

**DOI:** 10.3390/microorganisms11081941

**Published:** 2023-07-29

**Authors:** Md Shakhawat Hossain, Christine Frith, Siddhartha Shankar Bhattacharyya, Paul B. DeLaune, Terry J. Gentry

**Affiliations:** 1 Department of Soil and Crop Sciences, Texas A&M University, College Station, TX 77843, USA; 2 Texas A&M AgriLife Research, College Station, TX 77843, USA; 3 Department of Geosciences, Texas A&M University, College Station, TX 77843, USA; 4 Texas A&M AgriLife Research, Vernon, TX 76384, USA

**Keywords:** nodules, PGP, *Rhizobia*, bacterial endophytes, legumes, peanut

## Abstract

It is evident that legume root nodules can accommodate rhizobial and non-rhizobial bacterial endophytes. Our recent nodule microbiome study in peanuts described that small nodules can harbor diverse bacterial endophytes. To understand their functional role, we isolated 87 indigenous endophytes from small nodules of field-grown peanut roots and characterized them at molecular, biochemical, and physiological levels. The amplified 16S rRNA genes and phylogenetic analysis of these isolates revealed a wide variety of microorganisms related to the genera *Bacillus*, *Burkholderia*, *Enterobacter*, *Herbaspirillum*, *Mistsuaria*, *Pantoea*, *Pseudomonas*, and *Rhizobia*. It was observed that 37% (100% identity) and 56% (>99% identity) of the isolates matched with the amplified sequence variants (ASVs) from our previous microbiome study. All of these isolates were tested for stress tolerance (high temperature, salinity, acidic pH) and phosphate (P) solubilization along with ammonia (NH_3_), indole-3-acetic acid (IAA), 1-aminocyclopropane-1-carboxylate deaminase (ACCD), and siderophore production. The majority (78%) of the isolates were found to be halotolerant, thermotolerant, and acidophilic, and a few of them showed a significant positive response to the production of IAA, NH_3_, siderophore, ACCD, and P-solubilization. To evaluate the plant growth promotion (PGP) activity, plant and nodulation assays were performed in the growth chamber conditions for the selected isolates from both the non-rhizobial and rhizobial groups. However, these isolates appeared to be non-nodulating in the tested conditions. Nonetheless, the isolates 2 (*Pantoea*), 17 (*Burkholderia*), 21 (*Herbaspirillum*), 33o (*Pseudomonas*), and 77 (*Rhizobium* sp.) showed significant PGP activity in terms of biomass production. Our findings indicate that these isolates have potential for future biotechnological applications through the development of biologicals for sustainable crop improvement.

## 1. Introduction

Legume nodules are specialized structures that form on the roots of leguminous plants via the symbiotic relationship with nitrogen-fixing soil bacteria called rhizobia. The main function of these nodules is to facilitate the conversion of atmospheric nitrogen gas (N_2_) into plant-available forms such as ammonium (NH_4_^+^) or nitrate (NO_3_^−^) through a process known as biological nitrogen fixation (BNF) [1]. Traditionally, it was believed that legume nodules exclusively housed *Rhizobium*/*Bradyrhizobium* as a host symbiont, but recent studies have shown that other non-rhizobial bacterial endophytes (NRBEs) coexist with rhizobial strains [2,3,4]. NRBEs can enter host plants by colonizing the root’s inner cells/tissues or by exploiting discontinuities on the plant’s surface. Once inside, they establish a symbiotic relationship with the host and receive a protected and nutrient-rich environment in exchange for improving the plant’s health [3,4]. The coexistence of rhizobia and NRBEs in legume nodules highlights the complexity of the nodule microbiome and the interactions that occur within this environment.

Both rhizobia and NRBEs from nodules have been isolated from various legume species including wild legumes, fenugreek, lupine, groundnut, peanut, etc. [4,5,6,7,8,9]. Several genera of NRBEs other than rhizobia were isolated including *Agrobacterium*, *Bacillus*, *Enterobacter*, *Paenibacillus*, *Erwinia*, *Aerobacter*, *Herbaspirillum*, *Klebsiella*, *Pantoea*, and *Pseudomonas* [10,11]. However, the functional roles of NRBEs in legumes have not been fully explored [12].

Previous studies have shown that NRBEs can directly or indirectly influence PGP activity by producing indole-3-acetic acid (IAA), siderophores, 1-aminocyclopropane-1-carboxylate deaminase (ACCD), and P-solubilization [5,12]. Tryptophan, an amino acid, serves as a precursor molecule for IAA biosynthesis [13], and IAA is an essential phytohormone for plant growth and development including cell elongation, root formation, etc. Some IAA-producing NRBEs include *Enterobacter* spp. and *Pseudomonas* spp. [13], which can improve the seed germination, shoot growth, and root architecture in legumes [14]. Siderophores (molecules) attach to available forms of iron (e.g., Fe^3+^), allowing for an increased solubilization and accessibility of Fe [15,16]. In addition, the release of the ACCD enzyme leads to the cleavage of ACC, which is a precursor of ethylene, resulting in the production of ammonia (NH_3_) and α-ketobutyrate. This process reduces the levels of ethylene in plants and enhances their resistance to environmental stress [17].

Again, NRBEs can facilitate nutrient acquisition (e.g., P-solubilization) by producing organic acids [18]. Ferchichi et al. [6] observed that the efficiency of P-solubilization by bacterial endophytes obtained from lupine nodules varied. Among these, *Pseudomonas brenneri* exhibited the highest P-solubilization efficiency, followed by *Bacillus subtillis* and *Burkholderia gramini*, respectively. Recent studies elucidated that some NRBEs are capable of inducing stress tolerance, and some halotolerant NRBEs including *Brachybacterium* nov., *Zhihengliuella* sp., *Brevibacteriumcasei*, *Halomonas* sp., *Vibrio* sp., and *Pseudomonas* spp., have been isolated [19]. In addition, some halotolerant NRBEs from cowpea nodules, especially *Pseudomonas Fluorescens*, *Bacillus endophyticus*, *Bacillus pumilus*, and *Paenibacillus polymyxa* have shown potential for stimulating plant growth and development [20]. Ruiz-Díez et al. [21] postulated that NRBEs (e.g., *Phylobacterium myrsinacearum*), having the plant growth promoting ability, can withstand acidic pH. Currently, there is a dearth of knowledge on the presence of bacterial endophytes in the small nodules of peanuts and their potential ability to facilitate plant growth promotion and tolerate biotic and abiotic stresses.

Peanut (*Arachis hypogaea* L.) is an indigenous South American legume [22] and is widely grown in more than 100 countries [23]. Peanuts can form both effective and ineffective nodules with distinct sizes [24,25]. Large nodules (pink color, >1.5 mm) are effective for BNF, while small nodules (white color, <1.5 mm) are ineffective or exhibit lower nitrogen-fixing activity compared to medium-sized to large nodules [24]. Previously, the amplicon-based (16s rRNA) approach was used to study the peanut rhizosphere and bulk soil microbiome [26,27,28]. However, in our recent study on the peanut nodules’ microbiome [25], we showed that the small nodules (<1.5 mm) had approximately 31% of diverse sets of bacterial endophytes compared to the big nodules. But the function of these diverse NRBEs in the small nodules remains largely unexplored. To date, and to the best of our knowledge, no studies have been conducted to explore NRBEs from the small nodules of peanut roots specifically. Hence, in this study, the main objectives are to isolate and characterize mostly NRBEs together with a few examples of rhizobial isolates from small nodules of peanut roots and evaluate their role in PGP activity for biomass production.

## 2. Materials and Methods

### 2.1. Isolation of Bacterial Isolates from Small Nodules of Peanut Roots

To isolate bacterial endophytes, small nodules (<1.5 mm) from field-grown peanut roots (cultivated at the Texas A&M AgriLife Research and Extension Center, Vernon) were used, which were harvested during our previous study [25]. Twenty-four small nodules (four from each plant) from peanut roots were collected and cleaned with water to remove any dirt or foreign particles attached to the nodules. Cleaned nodules were then surface sterilized using 70% ethanol for 1.5 min, washed with sterile water (H_2_O) three times, and then soaked in 10% commercial bleach for 5 min. After this, nodules were washed at least 5–6 times with sterile H_2_O to rinse off any trace element of bleach. Then, washed nodules were crushed in 1 mL of 1× sterilized phosphate-buffered saline, and the resulting suspension was streaked on yeast extract mannitol agar (YMA, catalog number M715; https://www.himedialabs.com/us/ (accessed on 27 August 2021)) plates with multiple dilution series. We also streaked 100 µL of the final washed solution from the above to check if any contamination occurred during the sterilization process. The streaked YMA agar plates were incubated at 28 °C for 3 to 5 days, and the selected isolates (pure colonies) were grown in liquid YMA and later archived in glycerol stock.

### 2.2. Identification and Molecular Characterization of Bacterial Isolates

The genomic DNA was extracted from all the selected isolates (87) using the Promega genomic isolation kit (https://www.promega.com/ (accessed on 15 July 2022)). The 16S rRNA gene was amplified from these isolates using 27F and 1492R primer sets [29] under the following PCR conditions: 1 cycle of 95 °C for 5 min (preheating), 25 cycles of 95 °C for 30 s, 55 °C for 30 s, 72 °C for 1 min, and a final extension period at 72 °C for 5 min. The amplified PCR products (5 µL) were loaded into 2% agarose gel to check the fragment size and then purified from the PCR products using the Promega PCR cleanup kit. The resulting purified PCR products were Sanger sequenced by Eton Bioscience Inc. (https://etonbio.com (accessed on 11 August 2022).

The 16S rRNA gene sequences of the isolates were compared with the 16S rRNA small subunit (SSA) using the SILVA database ACT (alignment, classification, and tree services) tools [30]. The annotated neighbor sequences were downloaded from the SILVA database and multiple sequence alignment was performed together with the peanut small nodule isolate sequences using the MAFFT version 7 program [31]. For phylogenetic relationship analysis, the aligned Clustal data were opened in ClustalX and the Neighbor Joining (NJ) tree was constructed using the Jukes–Cantor model with bootstrap resampling = 1000 and visualized in FigTree. For the identification of isolates matched with peanut small nodule ASVs [25], the NCBI two-way blast was performed.

### 2.3. Biochemical Analysis of Isolates for Growth-Promoting Traits

#### 2.3.1. Ammonia (NH_3_) Production Assay

NH_3_ production assay was determined using the method described by Singh et al. [32] with some modifications. All the isolates were freshly grown in peptone water and incubated at 28 ± 2 °C for 48 to 72 h. After incubation, 0.5 mL of Nessler’s reagent was added to 4 mL of freshly cultured isolates in peptone water and incubated in the dark at room temperature for 15 to 30 min for color changes. An absorbance (450 nm) was then measured using a UV spectrophotometer. The NH_3_ production was calculated from three independent replications for each isolate and based on a standard curve (0–0.5 mg/mL) ammonium sulfate range (Figure S1A).

#### 2.3.2. Indole-3-Acetic Acid (IAA) Production Assay

To determine the amounts of IAA produced by each isolate, a colorimetric assay was performed with Van Urk Salkowski reagent using the method described by B. Mohite [33] with some modifications. All the isolates were freshly grown in YMA liquid medium and incubated at 28 °C for 4 days. After incubation, the culture broth was centrifuged, and equal volumes of isolate supernatant and Salkowski reagent (2% 0.5 M FeCl_3_ in 35% HClO_4_ solution) were added and incubated in the dark at room temperature. The absorbance (530 nm) was measured at 30 min and 120 min. The IAA amount was estimated from three independent replications for each isolate and compared with (5 mM) or without tryptophan added to the YMA medium. The IAA production was calculated using methods described by Gordon and Weber [34] and based on an IAA standard curve (0–30 µg/mL) concentration range (Figure S1B).

#### 2.3.3. P-Solubilization Assay (Agar Plate Method)

All the isolates were tested for P-solubilization using YMA and Pikovskaya’s (PVK) [35] agar plates containing known amounts of dipotassium phosphate (K_2_HPO_4_) and tricalcium phosphate (Ca_3_(PO_4_)_2_). Independent isolates were spotted four times on each plate, representing four replications, and incubated at 37 °C for 72 h. P-solubilization was observed for the development of clear zones surrounding the bacterial colony [36] and the P-solubilization index (PSI) was calculated according to the method by Pande et al. [37].

#### 2.3.4. Siderophore Production Assay

All the isolates were tested for siderophore production using the method described by Virpiranta et al. [38] with some modifications. Isolates were cultured in 3 mL fructose–glucose medium [38] and incubated at 28 °C for 72 h. After incubation, bacterial cultures were centrifuged, and equal volumes of bacterial supernatant and liquid Fe-CAS media [38] were mixed and then incubated in the dark at room temperature for 20 min. The absorbance (620 nm) was measured using a UV spectrophotometer, and siderophores produced by the isolates were calculated as percent siderophore units (PSUs) based on the formula used by Virpiranta et al. [38] from three independent replications for each isolate.

#### 2.3.5. ACCD Activity (Plate Assay)

All the isolates were screened for ACCD activity using the method described by Maheshwari et al. [39] with modified ACC medium containing 0.005% Bromothymol Blue and 0.008% Phenol Red. The isolates were cultured in YMA liquid medium at 28 °C for 48 h and then the bacterial pellets were washed twice with sterilized 0.1 M Tris-HCL (pH 7.5) via centrifugation. After washing, the bacterial cell pellets were spotted on modified Dworkin and Foster (DF) medium [40] with or without nitrogen (NH_4_SO_4_), and ACC (3 mM) was used only for the sole nitrogen source. Four independent spots were streaked on these plates for each isolate and incubated at 30 °C for 48 h to observe the color changes. ACCD catalysis was detected by color changes in the medium (due to change in pH), which catalyzes ACC into NH_3_ and ⍺-ketobutyrate.

#### 2.3.6. Tolerance of Isolates to High Temperature, NaCl, and Low pH

All the isolates were tested for growth at high temperature, elevated NaCl, and low pH on YMA agar plates. Freshly grown isolates were spotted four times as replications for a single isolate on YMA agar plates and incubated at 28 °C for 48 h for low pH (4, 5, 7) and 72 h for various NaCl concentrations (0, 0.15, 0.3, and 0.6 M). For high temperature, isolates were incubated at 37 °C, 40 °C, and 45 °C for 72 h. Bacterial growth was measured based on visible changes in growth and colony size on YMA agar plates. For example, when a colony became smaller in size by the effects of higher or lower dose/concentrations of above conditions, this was considered as slower or decreased growth, and no colony growth on the plate was counted as no growth.

### 2.4. Evaluation of Plant Growth Promotion (PGP) Ability with the Selected Endophytes

Peanut seeds var. Tamnut 74 (provided by Dr. John Cason, Texas AgriLife Research, Stephenville, TX, USA) were surface sterilized using 70% EtOH for 2 min and were rinsed with sterilized H_2_O three times before being immersed with 10% commercial bleach solution for 8 min. This was followed by 6–7 vigorous washes with sterilized water to rinse off any trace elements of bleach. Four to five seeds were then germinated in plastic pots containing sterilized coarse vermiculite and perlite (PVP industries, North Bloomfield, OH, USA) mixture (3:1 *w*/*w*) and grown on growth racks at 25–26 °C with 16 h light and 8 h dark conditions. The following three treatments were used: (1) negative control without any nitrogen source; (2) positive control supplemented with 0.5 mM NH_4_NO_3_; and (3) inoculation with isolates + 0.5 mM NH_4_NO_3_. The N source was applied to the plants once a week. The selected isolates were freshly cultured at 28 °C for 48 h, centrifuged, and washed with sterile water before inoculation. The germinated peanut seedlings at the base of tap roots were inoculated with 3–4 mL of freshly prepared inoculum for each pot (concentration adjusted to 0.2, OD 600 nm), and seven to twelve total plants from 2–3 pots were used for data analysis and served as replications for each isolate.

## 3. Results

### 3.1. Isolation of Bacterial Endophytes from Small Nodules of Peanut Roots

The bacterial endophytes were isolated from a mix of small nodules of peanut roots. A total of 87 isolates were randomly selected based on the morphology, appearance, shape, diameter, and color of the colonies on the YMA agar plates. No isolates were grown or observed on the YMA agar plates when the sterilized water from the final wash of the nodule surface was used as an inoculum. The isolates were given an ID name (numerical order) for the subsequent molecular identification, biochemical characterization, and plant growth promotion assay.

### 3.2. Molecular Identification of Small Nodule Endophytes of Peanut Roots

The 16S rRNA gene sequences from all the isolates were analyzed against the 16S rRNA small subunit (SSA) using ACT tools from the SILVA database (see Section 2), which provides sequence alignment, taxonomic classification, and phylogenetic tree services. Based on the percentage of identity in the ACT analysis, all 87 isolates were characterized at the family and genus levels (Figure S2, Table S1). To further understand and identify the sequence similarity of these isolates with previously published microbiome sequences of peanut nodules [25], an NCBI two-way blast analysis was used and it was found that 37% (*n* = 32) and 56% (*n* = 49) of isolates were 100% and >99% identical with the ASVs, respectively (Table 1 and Table S1).

**Figure 1:**
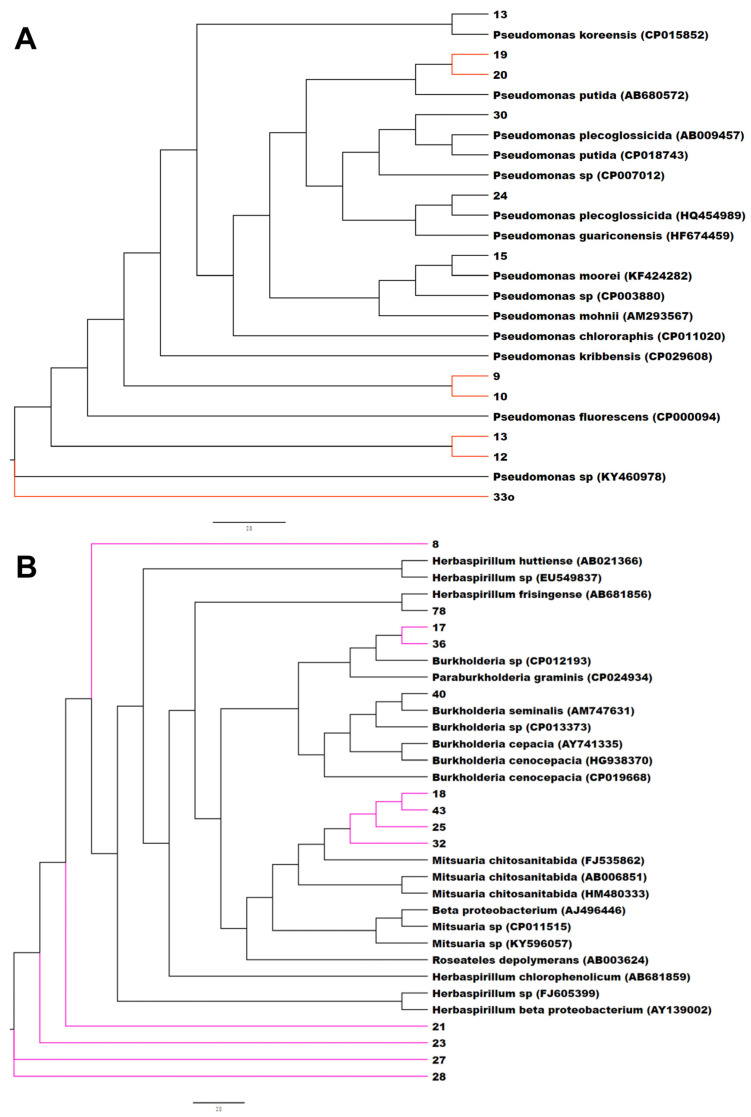

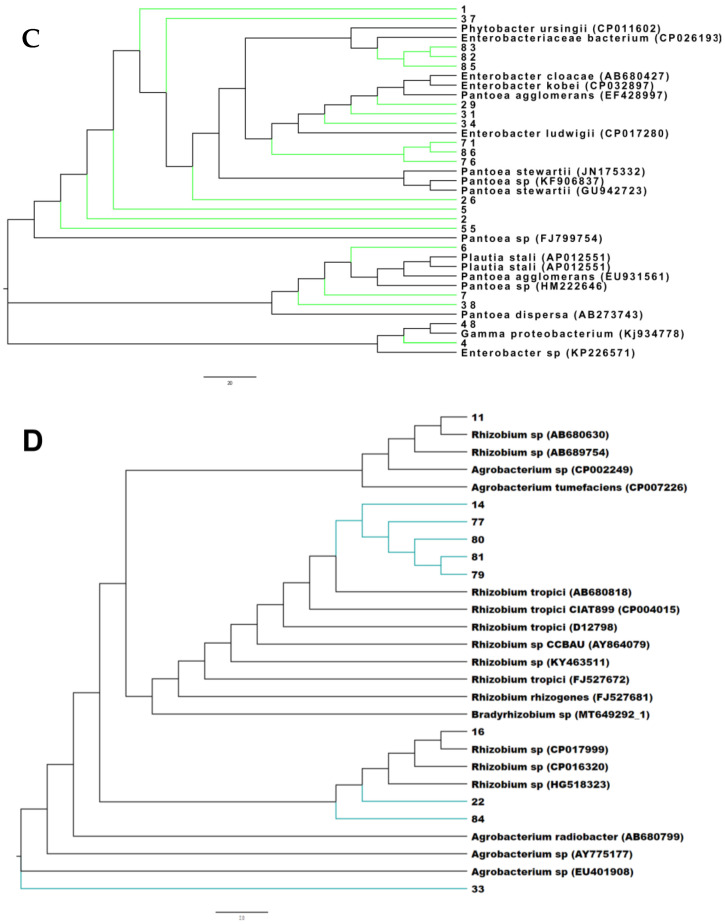
Phylogenetic tree based on 16S rRNA gene sequences of small nodule isolates of peanut roots with known neighbor sequences from SILVA database. The accession numbers of neighbor sequences are shown after the genus/species names. (**A**) *Pseudomonas*, (**B**) *Herbaspirillum* and *Burkholderia*, (**C**) *Enterobacter* and *Pantoea*, and (**D**) *Rhizobium* clades are shown. Colored branches indicate distinct/novel isolates from small nodules of peanut roots.

The phylogenetic analysis using all the isolates with reference neighboring sequences from the SILVA database shows that the peanut small nodule endophytes were grouped among several branches and nodes with the genera *Enterobacter* and *Pantoea*, *Pseudomonas*, *Herbaspirillum* and *Burkholderia*, *Rhizobium*, *Flavobacterium*, *Streptomyces*, and *Bacillus* (Figure S2). A close analysis of the phylogenetic relationship of each of these branches clearly showed that the small nodule endophytes of peanut plants were unique, made several nodes, and were closely grouped together with the genera *Enterobacter* and *Pantoea, Herbaspirillum* and *Burkholderia*, *Pseudomonas*, *Bacillus*, and *Rhizobium* (Figure 1 and Figure S3).

### 3.3. Characterization of Growth Promoting Traits of Small Nodule Endophytes from Peanut Roots

After molecular characterization, we lost four isolates due to growth failure during storage. All 83 of the remaining isolates were tested at least two times with 3–4 replications (for a single isolate) for thermotolerance, halotolerance, acid tolerance conditions, and growth promoting traits, i.e., NH_3_, IAA, and siderophore production; ACCD activity; and P-solubilization.

For the high temperature tolerance tests (37 °C, 40 °C, and 45 °C), all 83 isolates were grown normally at 37 °C (Table S2). With the rise in temperature, the number of isolates and their growth decreased or slowed down (e.g., 33% at 45 °C). For the salinity and low pH tests, all the isolates were grown at 28 °C (Table S2) with various NaCl concentrations (0, 0.15, 0.3, and 0.6 M) and pH levels (4, 5, 7), respectively. Without NaCl or with a low concentration of NaCl (0.15 M), the isolates were grown normally. In contrast, higher concentrations of NaCl (0.3 and 0.6 M) or a low acidic pH (4) seemed to have no significant impact on the endophytes’ growth. Our data suggest that most of the isolates can tolerate a higher salt concentration and a low acidic pH. Moreover, a select few can also tolerate higher temperature conditions.

**Figure 2:**
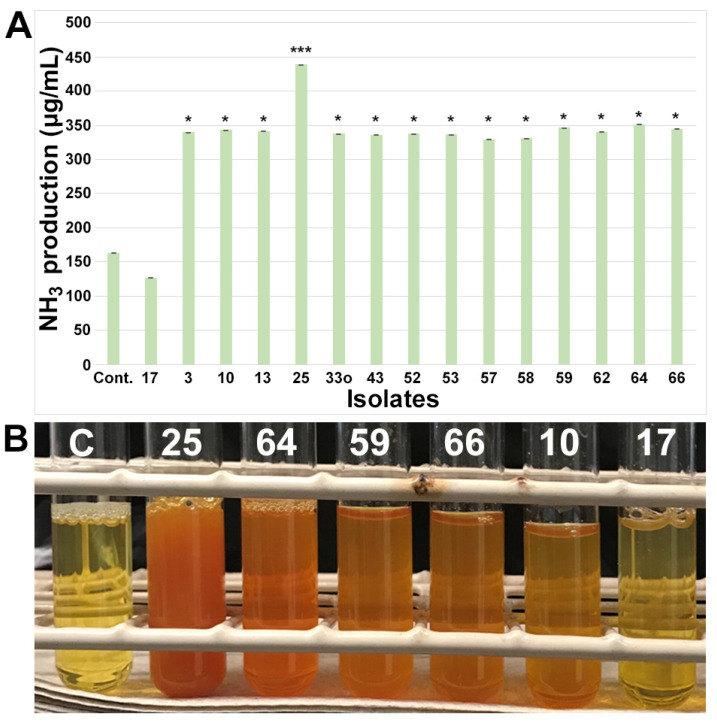
Screening and quantification of ammonia (NH_3_) production in small nodule isolates from peanut roots. (**A**) NH_3_ production assay performed in peptone water liquid medium and quantified via absorbance after incubation with Nessler’s reagent in dark conditions, and production of NH_3_ was estimated based on NH_4_SO_4_ standard curve (Figure S3A). Isolate names are indicated on the x-axis. The values are the means of three replicates, and the bars indicate ± standard error. The significance levels are indicated by *p* < 0.05 = *; *p* < 0.001 = ***. (**B**) The representative of qualitative colorimetric NH_3_ assay showing a subset of isolates from small nodule of peanut roots as indicated by the numerical number. ‘Cont.’ in A and ‘C’ in B stands for media control and the numerical number indicates isolate number.

In terms of NH_3_ production, 16% (*n* = 14) of isolates showed significant (*p* < 0.05) NH_3_ production (Figure 2) compared to the media control and the negative NH_3_ producer *Burkholderia* (isolate 17). NH_3_ production (300–350 µg/mL) was observed in only three groups of bacteria (*Bacillus*, *Pseudomonas*, and *Mitsuaria*) and remained consistent among most of the isolates except for isolate 25 (*Mitsuaria*), which had a significant (*p* < 0.001) amount of NH_3_ production (around 400 µg/mL).

**Figure 3:**
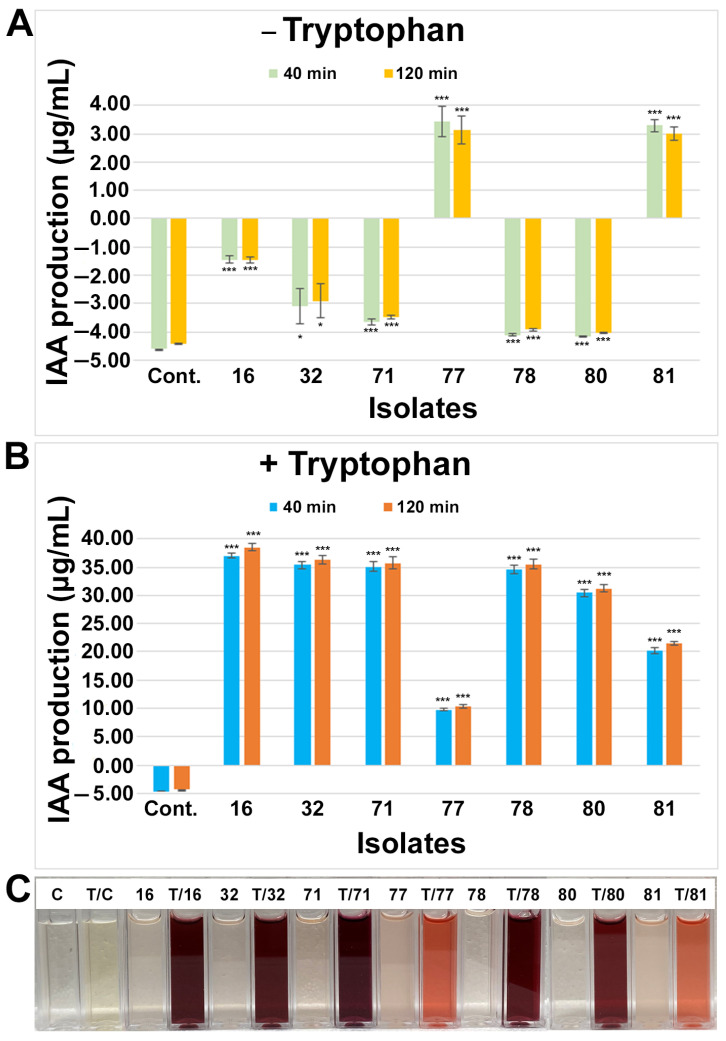
Screening and quantification of indole 3-acetic acid (IAA) production from small nodule isolates of peanut roots without (**A**) or with (**B**) tryptophan. Isolate names are indicated on the x-axis. IAA production assay was performed in the liquid medium, quantified absorbance was performed after 40 min and 120 min incubation with Salkowski reagents in dark conditions, and production of IAA was estimated based on IAA standard curve (Figure S3B). The values are the means of three replicates and the bars indicate ± standard error. The significance levels are indicated by *p* < 0.05 = *; *p* < 0.001 = ***. ‘Cont.’ in (**A**,**B**) stands for media control, and the numerical number indicates isolate number. (**C**) Qualitative colorimetric IAA assays after 40 min from small nodule isolates of peanut roots shown in (**A**,**B**). ‘C’ stands for media control, ‘T’ stands for tryptophan-treated assay, and the numerical number indicates corresponding isolate number in (**A**,**B**).

A Salkowski colorimetric assay was performed (see Materials and Methods; Figure 3C) to test and identify the IAA-producing endophytes. Based on the assay, the IAA production was calculated using the standard curve (Figure S1B), and seven isolates were identified as IAA-producing endophytes (Figure 3). Among them, most of the isolates, including the media control, showed a negative IAA production, while two rhizobial categories of the isolates (77 and 81) significantly (*p* < 0.001) produced IAA at the level of 3–4 µg/mL without any tryptophan application to the growth media. All seven isolates from the genera *Rhizobium* (16, 77, 80, 81), *Herbaspirillum* (78), *Mitsuaria* (32), and *Pantoea* (71) showed significant (*p* < 0.001) levels of IAA production when tryptophan was supplemented with the media, suggesting that these isolates required tryptophan to produce or increase further IAA production. After adding tryptophan to the media, IAA production was increased 3–6× by the isolates 77 and 81, while isolates 16, 32, 71, 78, and 80 produced 7–26× more IAA. Since Salkowski reagent reacts to IAA by changing color within minutes, and the respective intensity and concentration may continue to increase for a short period of time, we observed IAA production after 40 min and 120 min. The IAA production did not significantly change by the extended period with or without tryptophan.

To screen for ACCD-producing endophytes in our collection of small nodule endophytes of peanut roots, we tested all the isolates for ACCD activity. Among the 83 isolates that we tested, only 3 isolates (8, 21, and 28) belonging to the genus *Herbaspirillum* showed strong growth on the DF medium supplemented with ACC with two separate indicator dyes (bromothymol blue and phenol red) compared with or without a N source (Table S3; Figure S4), suggesting that these isolates were solely utilizing ACC as a N source in the production of NH_3_ and ⍺-ketobutyrate.

**Figure 4:**
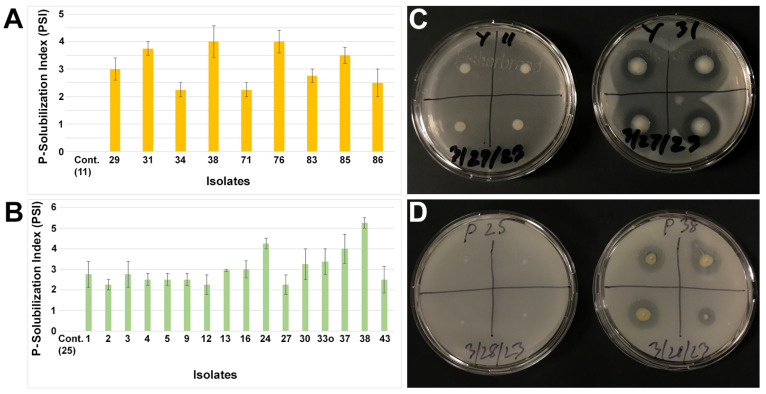
Phosphate solubilization index (PSI) from small nodule isolates of peanut roots using YMA (**A**) and PVK (**B**) media. The values are the means of four replicates and the bars indicate ± standard error. ‘Cont.’ in (**A**,**B**) stands for negative P-solubilization control for the indicated isolates and the numerical number indicates isolate number. Representative agar plate assay for P-solubilization on YMA (**C**) and PVK (**D**) from small nodule isolates of peanut roots.

Endophytic bacteria also play a significant role in plant growth promotion by P-solubilization. We investigated all small nodule endophytes of peanut roots to test P-solubilization using two types of media (Figure 4). In the media constituents, YMA had a known amount of dipotassium phosphate, whereas PVK contained tricalcium phosphate. The P-solubilization index (PSI) was seen in distinct groups of isolates based on their media types. Considering PSI > 2.0, only nine isolates were shown to have P-solubilization activity due to the YMA media; however, that number was increased to almost 50% (*n* = 16 isolates) when using the PVK media. No P-solubilization was observed for the control isolates 11 and 25 for the YMA and PVK media, respectively. The isolates from the *Enterobacteriaceae* family were mostly abundant for P-solubilization by the YMA media, whereas the isolates from the genera *Pseudomonas* and *Pantoea* predominantly solubilized P on the PVK media. Only the bacteria from the *Pantoea* group were observed to undergo P-solubilization on both the YMA and PVK media, and a single isolate (38) showed the highest amount of PSI activity when using these two media types. In addition to the genera *Pseudomonas* and *Pantoea*, a single isolate from *Rhizobium* (16), *Herbaspirillum* (27), and *Mitsuaria* (43) were also observed to have PSI activity due to the PVK media. However, no PSI activity was observed in these groups of bacteria when the YMA media was used.

**Figure 5:**
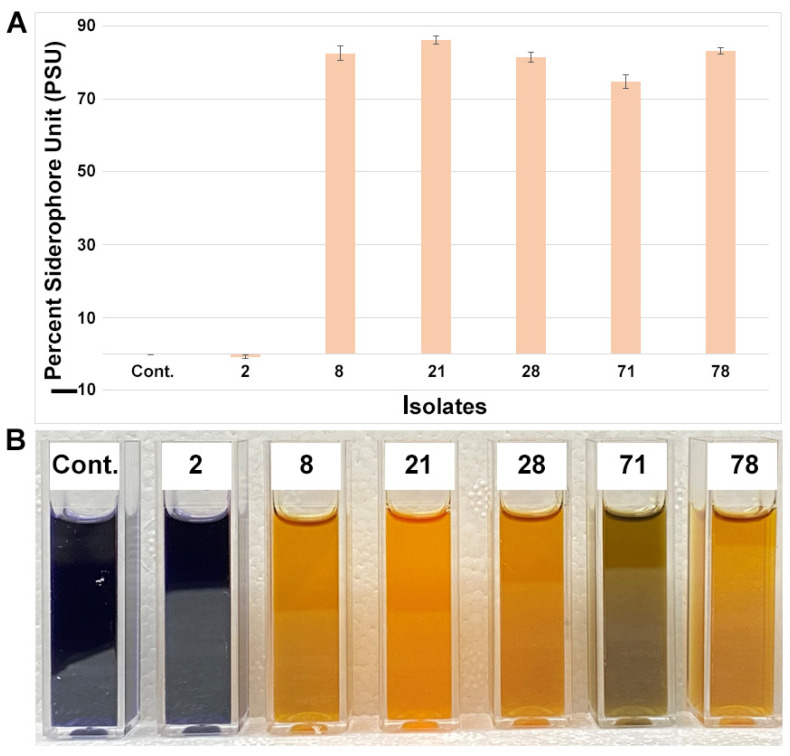
Quantitative and qualitative analyses of percent siderophore production from small nodule isolates of peanut roots. (**A**) Average siderophore production was calculated as percent siderophore unit (psu). The values are the means of three replicates and the bars indicate ± standard error. (**B**) Qualitative colorimetric siderophore assays from small nodule isolates of peanut roots shown in (**A**). ‘Cont.’ stand for media control and the numerical number indicates corresponding isolates number in A.

To investigate siderophore-producing endophytes, we tested all the isolates using liquid Fe-CAS medium. As evidence for siderophore production, microbial isolates normally alter the color from dark blue to yellow/orange in liquid CAS medium (Figure 5). Five endophytic small nodule isolates were found to be strong siderophore producers compared to the media control and the negative siderophore producer *Pantoea* (isolate 2). It was observed that most of them were from the *Herbaspirillum* group of bacteria. However, a positive siderophore-producing isolate (71) from the *Pantoea* group was also observed, suggesting variable siderophore production within the same groups of bacteria.

### 3.4. Plant Growth Promotion (PGP) Assay

**Figure 6:**
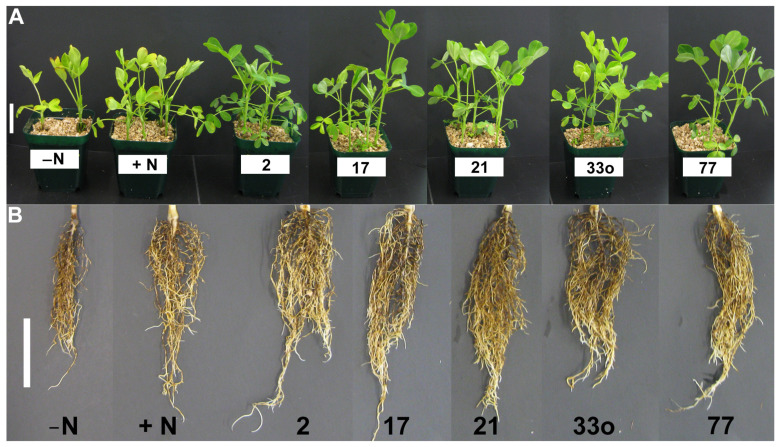
Plant growth promotion (PGP) assays using small nodule endophytic isolates from peanut roots as indicated by the numerical number. (**A**) Peanut plants and shoot growth after 24 days post inoculation (dpi) with (+N) or without (−N) N source. (**B**) Images of peanut root growth from the corresponding plants in (**A**).

**Figure 7:**
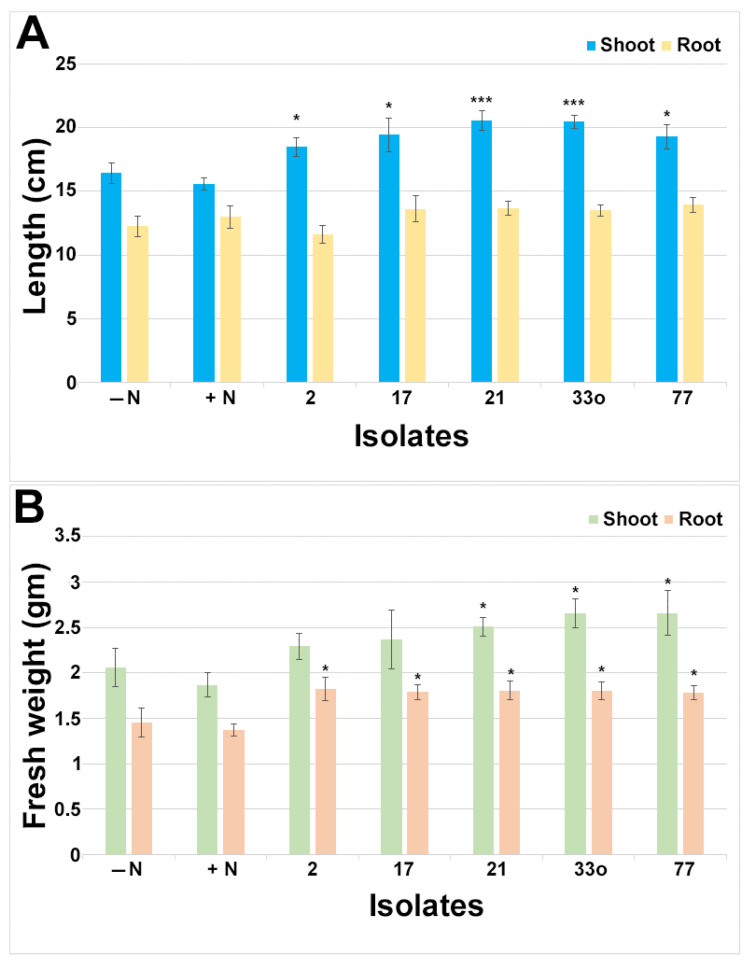
Effect of the shoot and root growth induction by the small nodule endophytes from peanut roots. The shoot and root length (**A**) and fresh weight (**B**) were measured after 24 days post inoculation with or without added N source. The bar indicates ± standard error from 7 to 12 individual plants for shoot and root length and fresh weight. Student’s t-test was conducted based on N-fed plants’ shoots and roots as controls, and the significance levels are indicated by *p* < 0.05 = *; *p* < 0.001 = ***.

To investigate the endophytes’ roles in peanut growth promotion, isolates 2, 17, 21, 25, 33o, 64, and 77 (*Pantoea*, *Burkholderia*, *Herbaspirillum*, *Mitsuaria*, *Pseudomonas*, *Bacillus*, and *Rhizobium* sp., respectively), were inoculated from non-rhizobial and rhizobial groups on pre-germinated peanut seedlings along with non-inoculated seedlings with N (+control) and/or without a N (−control) source (Figure 6, Figure 7 and Figure S5). At 24 days post inoculation, isolates 2 (*Pantoea*), 17 (*Burkholderia*), 21 (*Herbaspirillum*), 33o (*Pseudomonas*), and 77 (*Rhizobium* sp.) showed healthy plants with visible green shoots compared to the non-inoculated seedlings without N or even compared with the N-fed seedlings (Figure 6A), suggesting shoot biomass improvement via the application of these isolates. Similarly, root biomass induction was observed by these corresponding isolates (Figure 6B) after the soil debris was rinsed off. However, isolates 25 (*Mitsuaria*) and 64 (*Bacillus*) did not show noticeable plant biomass induction, although it seemed that isolate 25 might have a slightly increased shoot biomass (Figure S5). The quantitative analysis for the biomass of the shoots and roots via the application of these isolates clearly showed significant (*p* < 0.05) growth promotion compared to the seedlings without inoculation and N sources (Figure 7). Although all five isolates had significant (*p* < 0.05) shoot length induction, the quantitative fresh weight analysis revealed that only three of them (21, 33o, and 77) significantly matched with the shoot length (*p* < 0.05). Although no significant differences were observed for the root length, the root fresh weight was significantly increased by these five isolates (*p* < 0.05).

## 4. Discussion

Previously, bacterial endophytes were characterized from field-grown peanut seeds, cotyledons and germs, and the root tips of seedlings [41,42,43]. However, the isolation and characterization of bacterial endophytes from peanut small nodules remain largely unknown. In this study, we isolated and identified 87 indigenous bacterial endophytes from small nodules of field-grown peanut roots. The molecular characterization using the SILVA database and NCBI revealed that diverse bacterial endophytes inhabited the small nodules of peanut roots including *Rhizobium* and NRBEs. Among these, four isolates were lost during storage due to growth failure in the culture medium. We randomly selected bacterial isolates on the YMA agar plates without selecting any antibiotic or indicator dyes, and a further analysis of the 16S rRNA genes and their taxonomic classifications revealed that *Bacillaceae* was the predominant group of endophytes, followed by *Erwiniaceae*, *Pseudomonadaceae*, *Rhizobiaceae*, *Enterobacteriaceae*, *Oxalobacteraceae*, *Comamonadaceae*, *Burkholderiaceae*, *Flavobacteriaceae*, and *Streptomycetaceae*. *Bacillaceae* was also observed as the dominant group in the previous studies where isolates were obtained from peanut seeds, root tips, or from cotyledons and germs [41,42,43]. The phylogenetic relationship analysis revealed that most of the endophytes isolated from the small nodules of peanut plants clustered together and generated unique nodes and branches. This is consistent with our previous study on the peanut nodule microbiome, where we observed a diverse set of endophytic microbes that were abundantly present in the small nodules of peanut roots compared to the big nodules [25]. It was also previously reported that *Pseudomonas*, *Enterobacter*, and *Klebsiella* were also isolated from the nodules of peanut roots [8]. The coexistence of diverse NRBEs in the nodules is not new [42], and NRBEs have been isolated from many legumes [44,45,46,47].

Numerous previous studies have reported that NRBEs can mitigate adverse impacts of climate change and soil stresses such as drought, salinity, acidity, alkalinity, and heavy metal toxicity [12,48,49]. With the aforementioned considerations in mind, we conducted a comprehensive evaluation of our endophyte collections to identify the strains that exhibit resilience under conditions of high temperatures, low pH levels, and elevated concentrations of NaCl (indicative of salt stress). A significant proportion of the isolated endophytes demonstrated tolerance to acidic pH, high salinity, and elevated temperatures. While we have not yet tested these endophytes in real-world conditions, their potential as valuable biological resources for the development of formulations aimed at enhancing crop cultivation and sustainability and mitigating the adverse environmental impacts outlined above is apparent. Furthermore, a biochemical analysis of these endophytes was conducted to assess their capabilities in terms of NH_3_, IAA, and siderophore production; ACCD activity; and P-solubilization. A subset of endophytes from each category exhibited these traits, indicating their potential to promote plant growth. This is supported by numerous previously published studies [49,50,51,52,53,54].

The PGP activity (especially morphology and biomass) was observed by interacting with several of these endophytes from non-rhizobial and rhizobial groups in the peanut roots under growth chamber conditions. Not all of the tested endophytes (such as 25, 64) showed growth promoting traits when interacting with peanut plants; however, isolates 2, 17, 21, 33o, and 77 from *Pantoea*, *Burkholderia*, *Herbaspirillum*, *Pseudomonas*, and *Rhizobium* showed significant growth promotion for biomass production. In this study, we observed that *Pantoea* sp. performed better growth promotion in peanut; however, Li et al. [43] found that *Pantoea dispersa* caused peanut plants to wilt, suggesting that species variations can function differently. All of these endophytes appeared to be non-nodulating under the tested growth chamber conditions. Nonetheless, these endophytes consistently performed better biomass production. Overall, these findings shed light on the rich diversity of microorganisms within the small nodules of peanut roots and highlight the potential of non-rhizobial strains to positively influence peanut plant growth and development. The presence of these strains can improve stress tolerance, nutrient acquisition, and growth promotion. Further research in field settings may lead to the development of novel agricultural practices, such as targeted microbial inoculations, to enhance peanut productivity and sustainability. Harnessing the beneficial interactions between these endophytes and peanut plants can have significant implications for sustainable agriculture by reducing the reliance on chemical fertilizers and pesticides while enhancing crop productivity and resilience in the face of changing environmental conditions.

## Figures and Tables

**Table 1:** Taxonomic characterization of 32 small nodule isolates of peanut roots that were 100% matched with amplicon sequence variants (ASVs) using the NCBI two-way blast. The blast was conducted using 16S-rRNA gene sequences, and the isolates’ fragment sizes (bp) and annotated taxa (genus) information are shown based on SILVA database.

Isolate ID	% Identity with ASVs	ASV Fragment Size (bp)	Isolate Fragment Size (bp)	Genus
1	100.00	445	1107	*Pantoea*
2	100.00	445	1098	*Pantoea*
16	100.00	420	1145	*Allorhizobium-Neorhizobium-Pararhizobium-Rhizobium*
17	100.00	445	1195	*Burkholderia-Caballeronia-Paraburkholderia*
18	100.00	445	1117	*Mitsuaria*
22	100.00	420	1165	*Allorhizobium-Neorhizobium-Pararhizobium-Rhizobium*
23	100.00	445	1104	*Herbaspirillum*
25	100.00	445	1159	*Mitsuaria*
30	100.00	445	1113	*Pseudomonas*
32	100.00	445	1154	*Mitsuaria*
33	100.00	420	1140	*Allorhizobium-Neorhizobium-Pararhizobium-Rhizobium*
39	100.00	446	1118	*Bacillus*
42	100.00	446	1029	*Bacillus*
43	100.00	445	1004	*Mitsuaria*
49	100.00	446	1030	*Bacillus*
50	100.00	446	1022	*Bacillus*
56	100.00	446	1067	*Bacillus*
57	100.00	446	1075	*Bacillus*
58	100.00	446	1104	*Bacillus*
59	100.00	446	1087	*Bacillus*
60	100.00	446	1123	*Bacillus*
61	100.00	446	1029	*Bacillus*
62	100.00	446	1075	*Bacillus*
63	100.00	446	1053	*Bacillus*
64	100.00	446	1088	*Bacillus*
65	100.00	446	1071	*Bacillus*
66	100.00	446	1122	*Bacillus*
67	100.00	446	1095	*Bacillus*
68	100.00	446	1159	*Bacillus*
69	100.00	446	1095	*Bacillus*
77	100.00	420	1143	*Rhizobium* sp.
84	100.00	420	1232	*Rhizobium* sp. *CT 6-01*

## Data Availability

Data will be available upon request to the corresponding author.

## Supplementary Materials

The following supporting information can be downloaded at: https://www.mdpi.com/article/10.3390/microorganisms11081941/s1. Figure S1. Preparation of the standard curve concentrations. (A) NH_4_SO_4_ and (B) IAA for the estimation of NH_3_ and IAA production by spectrophotometric absorbances. Figure S2. Phylogenetic relationship of 87 small nodule isolates of peanut roots along with known neighbor sequences from SILVA database. Phylogenetic tree was made based on 16S rRNA gene sequences. The accession number of neighbor sequences are shown after the genus/species name. Figure S3. Phylogenetic relationship of *Bacillus* strains from small nodules of peanut roots with neighbor sequences from SILVA database. The dendrogram was made based on 16S rRNA gene sequences. The accession number of neighbor sequences are shown after the genus/species name. Colored line/branches indicate distinct/novel isolates from peanut small nodules. Figure S4: Peanut small nodule isolates 28 show aminocyclopropane-1-carboxylate deaminase (ACCD) activity. Figure S5. Plant growth promotion (PGP) assay using peanut small nodule endophytic isolates 25 (*Mitsuaria*) and 64 (*Bacillus*). Table S1. Taxonomic characterization of 87 small nodule isolates of peanut roots that were matched with amplicon sequence variants (ASV’s) using NCBI two-way blast. The blast was conducted using 16S rRNA gene sequences and the gene base pairs, annotated family & genus information are shown based on SILVA database. Table S2. Tolerance of small nodule isolates of peanut roots to high temperature, NaCl and pH on YMA agar plates. Table S3. Small nodule isolates of peanut roots show aminocyclopropane-1-carboxylate deaminase (ACCD) activity.
