# Supplementary material for: Isolation and Characterization of Bacterial Endophytes from Small Nodules of Field-Grown Peanut
Source: Microorganisms. 2023 Jul 29;11(8):1941. doi: 10.3390/microorganisms11081941 (PMC10458822; doi:10.3390/microorganisms11081941)
Supplement: Supplementary file 1 [file microorganisms-11-01941-s001.zip › Supplementary Information.pdf]

Supplementary Information:

Figure S1. Preparation of the standard curve concentrations. (A) NH_4SO_4 and (B) IAA for the estimation of NH_3 and IAA production by spectrophotometric absorbances.

Figure S2. Phylogenetic relationship of 87 small nodule isolates of peanut roots along with known neighbor sequences from SILVA database. Phylogenetic tree was made based on 16S rRNA gene sequences. The accession number of neighbor sequences are shown after the genus/species name.

Figure S3. Phylogenetic relationship of *Bacillus* strains from small nodules of peanut roots with neighbor sequences from SILVA database. The dendrogram was made based on 16S rRNA gene sequences. The accession number of neighbor sequences are shown after the genus/species name. Colored line/branches indicate distinct/novel isolates from peanut small nodules.

Figure S4: Peanut small nodule isolates 28 show aminocyclopropane-1-carboxylate deaminase (ACCD) activity.

Figure S5. Plant growth promotion (PGP) assay using peanut small nodule endophytic isolates 25 (*Mitsuaria*) and 64 (*Bacillus*).
