# Supplementary figures and images for: Isolation and Characterization of Bacterial Endophytes from Small Nodules of Field-Grown Peanut
Source: Microorganisms. 2023 Jul 29;11(8):1941. doi: 10.3390/microorganisms11081941 (PMC10458822; doi:10.3390/microorganisms11081941)

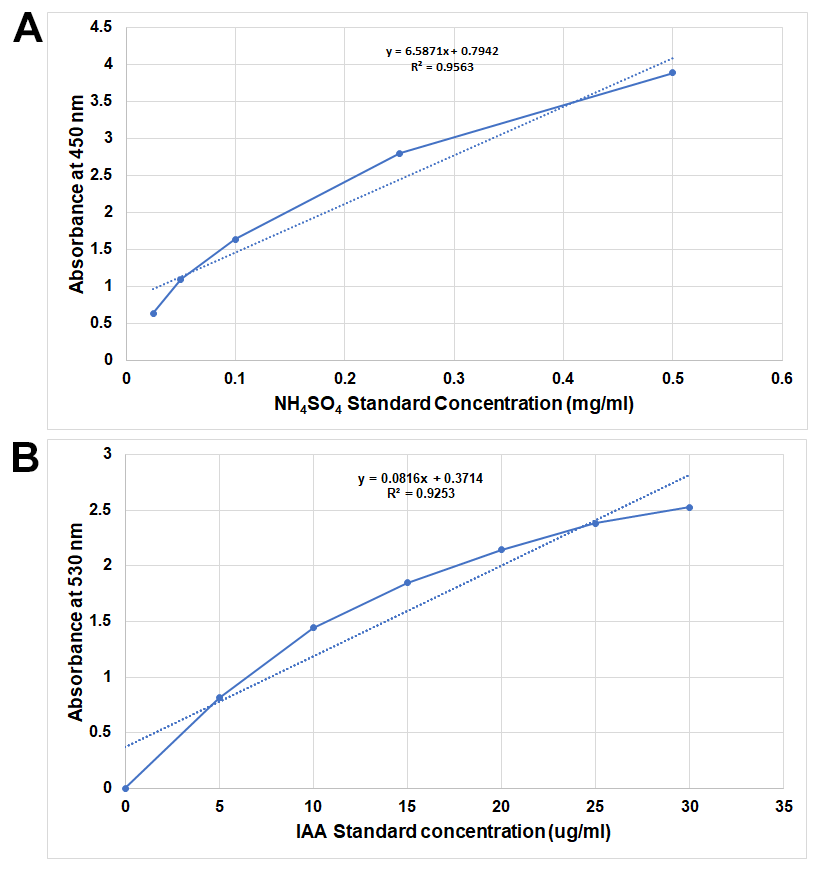

Supplement: Supplementary file 1 [file microorganisms-11-01941-s001.zip › Figure S1.tif]

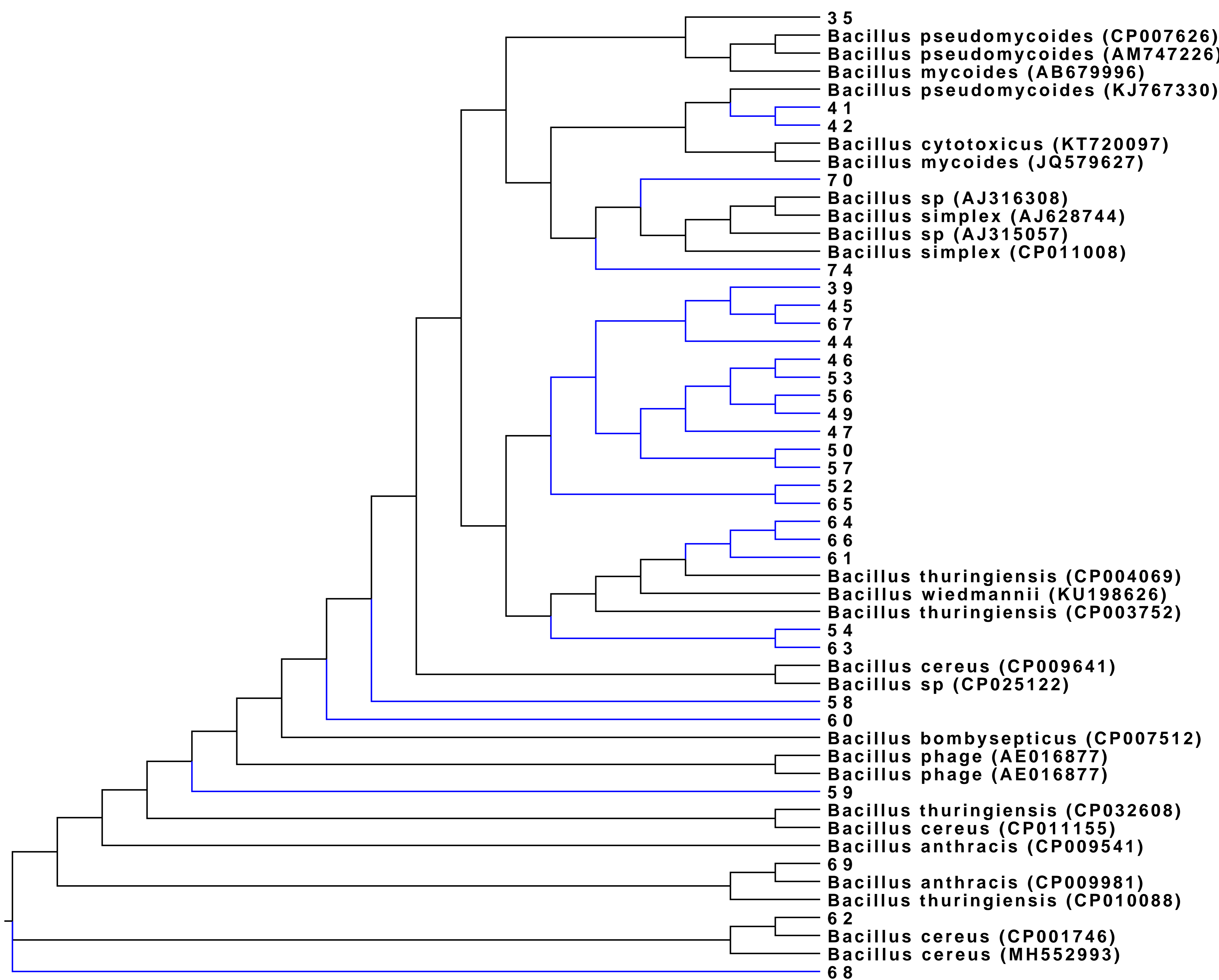

Supplement: Supplementary file 1 [file microorganisms-11-01941-s001.zip › Figure S3.pdf]

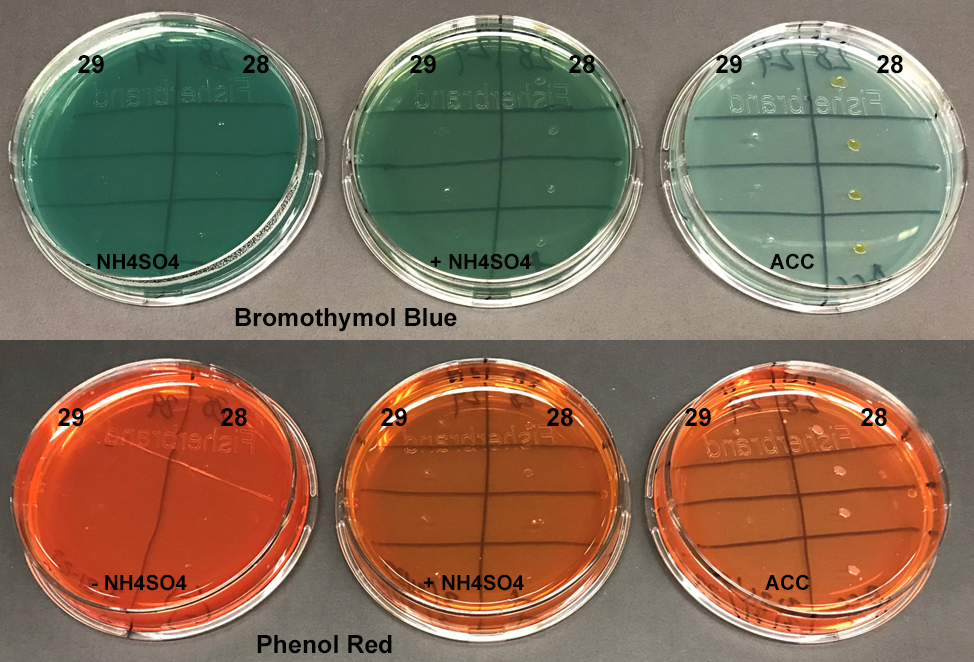

Supplement: Supplementary file 1 [file microorganisms-11-01941-s001.zip › Figure S4.tif]

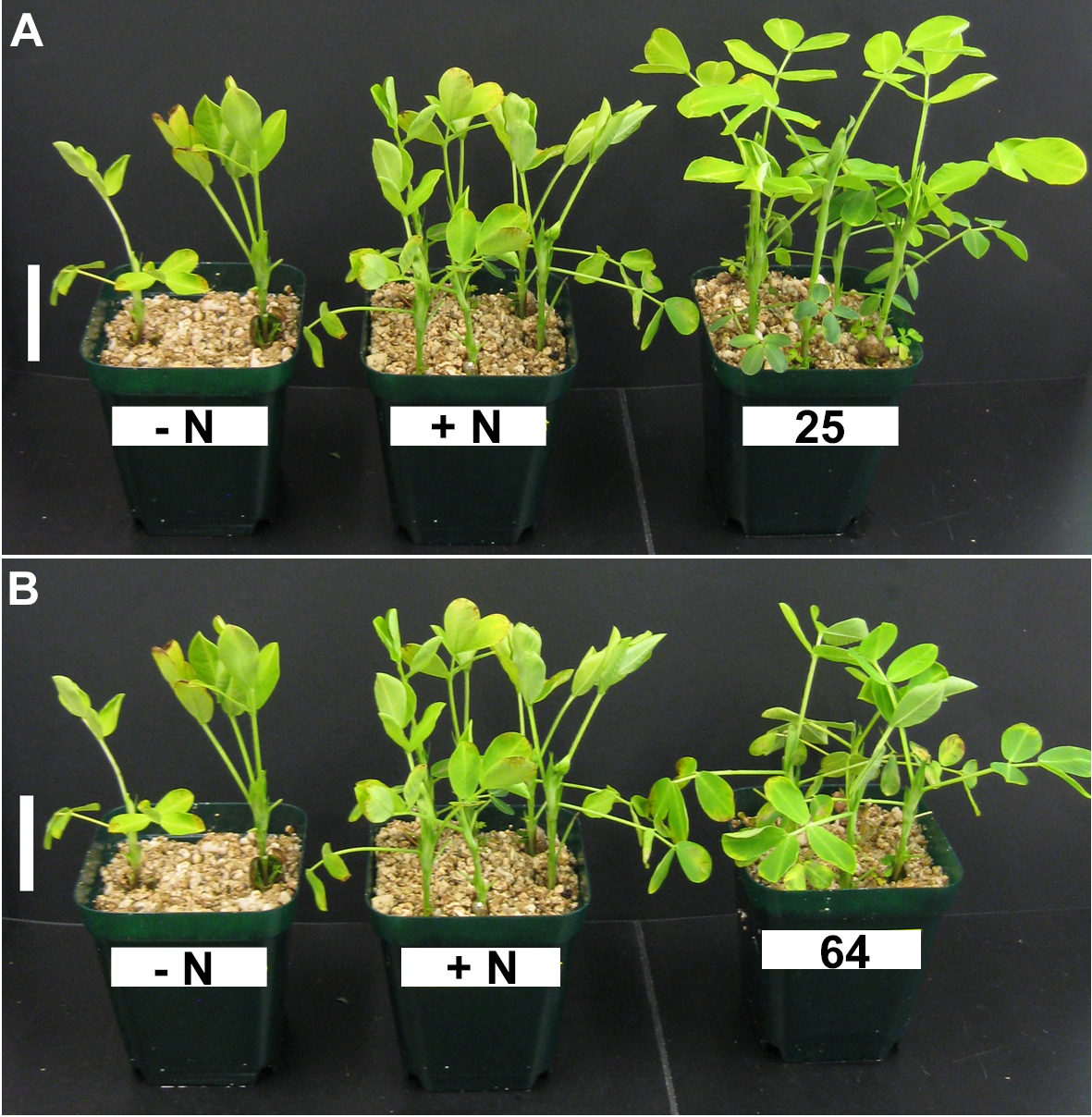

Supplement: Supplementary file 1 [file microorganisms-11-01941-s001.zip › Figure S5.tif]
