# Supplementary material for: Isolation and Characterization of Bacterial Endophytes from Small Nodules of Field-Grown Peanut
Source: Microorganisms. 2023 Jul 29;11(8):1941. doi: 10.3390/microorganisms11081941 (PMC10458822; doi:10.3390/microorganisms11081941)
Supplement: Supplementary file 1 [file Figure S2.pdf › Figure S2.pdf]

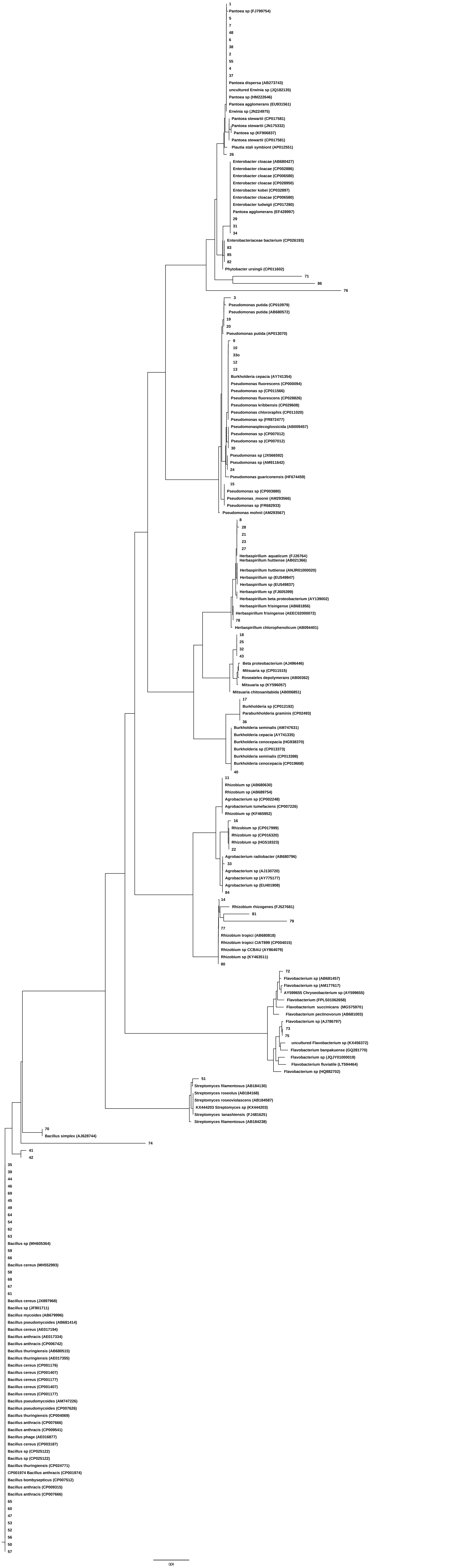
1
Pantoea sp (FJ799754)
5
7
48
6
38
2
55
4
37
Pantoea dispersa (AB273743)
uncultured Erwinia sp (JQ182135)
Pantoea sp (HM222646)
Pantoea agglomerans (EU931561)
Erwinia sp (JN224975)
Pantoea stewartii (CP017581)
Pantoea stewartii (JN175332)
Pantoea sp (KF906837)
Pantoea stewartii (CP017581)
Plautia stali symbiont (AP012551)
26
Enterobacter cloacae (AB680427)
Enterobacter cloacae (CP002886)
Enterobacter cloacae (CP006580)
Enterobacter cloacae (CP028950)
Enterobacter kobei (CP032897)
Enterobacter cloacae (CP006580)
Enterobacter ludwigii (CP017280)
Pantoea agglomerans (EF428997)
29
31
34
Enterobacteriaceae bacterium (CP026193)
83
85
82
Phytobacter ursingii (CP011602)
71
86
76
3
Pseudomonas putida (CP010979)
Pseudomonas putida (AB680572)
19
20
Pseudomonas putida (AP013070)
9
10
33o
12
13
Burkholderia cepacia (AY741354)
Pseudomonas fluorescens (CP000094)
Pseudomonas sp (CP011566)
Pseudomonas fluorescens (CP028826)
Pseudomonas kribbensis (CP029608)
Pseudomonas chlororaphis (CP011020)
Pseudomonas sp (FR872477)
Pseudomonasplecoglossicida (AB009457)
Pseudomonas sp (CP007012)
Pseudomonas sp (CP007012)
30
Pseudomonas sp (JX566592)
Pseudomonas sp (AM911642)
24
Pseudomonas guariconensis (HF674459)
15
Pseudomonas sp (CP003880)
Pseudomonas_moorei (AM293566)
Pseudomonas sp (FR682933)
Pseudomonas mohnii (AM293567)
8
28
21
23
27
Herbaspirillum aquaticum (FJ26764)
Herbaspirillum huttiense (AB021366)
Herbaspirillum huttiense (ANJR01000020)
Herbaspirillum sp (EU549847)
Herbaspirillum sp (EU549837)
Herbaspirillum sp (FJ605399)
Herbaspirillum beta proteobacterium (AY139002)
Herbaspirillum frisingense (AB681856)
Herbaspirillum frisingense (AEEC02000072)
78
Herbaspirillum chlorophenolicum (AB094401)
18
25
32
43
Beta proteobacterium (AJ496446)
Mitsuaria sp (CP011515)
Roseateles depolymerans (AB00362)
Mitsuaria sp (KY596057)
Mitsuaria chitosanitabida (AB006851)
17
Burkholderia sp (CP012192)
Paraburkholderia graminis (CP02493)
36
Burkholderia seminalis (AM747631)
Burkholderia cepacia (AY741335)
Burkholderia cenocepacia (HG938370)
Burkholderia sp (CP013373)
Burkholderia seminalis (CP013398)
Burkholderia cenocepacia (CP019668)
40
11
Rhizobium sp (AB680630)
Rhizobium sp (AB689754)
Agrobacterium sp (CP002248)
Agrobacterium tumefaciens (CP007226)
Rhizobium sp (KF465952)
16
Rhizobium sp (CP017999)
Rhizobium sp (CP016320)
Rhizobium sp (HG518323)
22
Agrobacterium radiobacter (AB680796)
33
Agrobacterium sp (AJ130720)
Agrobacterium sp (AY775177)
Agrobacterium sp (EU401908)
84
14
Rhizobium rhizogenes (FJ527681)
81
79
77
Rhizobium tropici (AB680818)
Rhizobium tropici CIAT899 (CP004015)
Rhizobium sp CCBAU (AY864079)
Rhizobium sp (KY463511)
80
72
Flavobacterium sp (AB681457)
Flavobacterium sp (AM177617)
AY599655 Chryseobacterium sp (AY599655)
Flavobacterium (FPLS01062658)
Flavobacterium succinicans (MG575970)
Flavobacterium pectinovorum (AB681003)
Flavobacterium sp (AJ786797)
73
75
uncultured Flavobacterium sp (KX456372)
Flavobacterium banpakuense (GQ281770)
Flavobacterium sp (JQJY01000019)
Flavobacterium fluviatile (LT594464)
Flavobacterium sp (HQ882702)
51
Streptomyces filamentosus (AB184130)
Streptomyces roseolus (AB184168)
Streptomyces roseoviolascens (AB184587)
KX444203 Streptomyces sp (KX444203)
Streptomyces tanashiensis (FJ481625)
Streptomyces filamentosus (AB184238)
70
Bacillus simplex (AJ628744)
74
41
42
35
39
44
46
69
45
49
64
54
62
63
Bacillus sp (MH605364)
59
66
Bacillus cereus (MH552993)
58
68
67
61
Bacillus cereus (JX897968)
Bacillus sp (JF901711)
Bacillus mycoides (AB679996)
Bacillus pseudomycoides (AB681414)
Bacillus cereus (AE017194)
Bacillus anthracis (AE017334)
Bacillus anthracis (CP006742)
Bacillus thuringiensis (AB680515)
Bacillus thuringiensis (AE017355)
Bacillus cereus (CP001176)
Bacillus cereus (CP001407)
Bacillus cereus (CP001177)
Bacillus cereus (CP001407)
Bacillus cereus (CP001177)
Bacillus pseudomycoides (AM747226)
Bacillus pseudomycoides (CP007626)
Bacillus thuringiensis (CP004069)
Bacillus anthracis (CP007666)
Bacillus anthracis (CP009541)
Bacillus phage (AE016877)
Bacillus cereus (CP003187)
Bacillus sp (CP025122)
Bacillus sp (CP025122)
Bacillus thuringiensis (CP024771)
CP001974 Bacillus anthracis (CP001974)
Bacillus bombysepticus (CP007512)
Bacillus anthracis (CP009315)
Bacillus anthracis (CP007666)
65
60
47
53
52
56
50
57
